# Ill Health from Over Eating

**Published:** 1887-09

**Authors:** 


### ILL HEALTH FROM OVER EATING.

It is doubtless true that the evils arising from intemperate eating, are quite as destructive as those which spring from the free use of liquors.

The former indulgence is prevalent among our better conditioned classes, with whom, what is termed a good dinner, is an every day affair.

Rich food, taken habitually in immoderate quantities, disorganizes the body and stupifies the brain.

Obesity is an unnatural condition, it is, in fact, a disease which impairs both body and brain. The case of Louis Cornaro is full of wise admonition :

Born at Venice about the year 1467, he was a man of weak constitution. Moreover, from the age of eighteen to that of thirty-five he pursued courses that would have seriously taxed the strongest constitution. Life at thirty-five was a burden to him, because of the disorders brought on by riotous living, and indulgence in every kind of excess. The next five years were passed in almost unremitted suffering. He was told by his physicians, when forty years old, that nothing could prolong his life for more than two or three years, but that such life as remained to him might be less painful than the years he had recently lived, if he would adopt more temperate habits. If ever there was a case where inherited constitution and an intemperate life threatened an early death, this was one. But, as events befell, it turned out that, if eve: there was a case where the life-preserving influence of wise regimen and abstemious habits was demonstrated, Cornaro's must be cited as especially significant.

At the age of forty, Cornaro began gradually to reduce the quantity of food, both liquid and solid, which he took each day, till at length he only took what nature absolutely required. He tells us that at first, he found this severe regimen very disagreeable, and confesses that "he relapsed from time to time to the flesh pots of Egypt." But, by resuming his efforts after each failure, he succeeded in less than a year, in adopting permanently a spare and moderate system. By this time he was already restored to perfect health. But thus far he had only followed the counsels of the physicians somewhat more steadily than they expected, or than is usual in such cases, and therefore, with unexpected good results. It was after he had recovered his health that he went on to those experiments, by which he seemed to show how life may be extended far beyond the Psalmist's allowance.

From temperance he proceeded to abstemiousness. Undeterred by the doubts of his physicians as to the wisdom of such a course, he diminished his daily allowance of food until at last, the yolk of an egg sufficed him for a meal! Throughout the time when he was thus reducing his allowance of food, his health and spirits kept improving. Nay, he tells us that even his enjoyment in eating had increased; for, he says, he could now get more pleasure from a small meal of dry bread, than he had ever obtained in the days of his excesses, from the most exquisite dainties of the table. As regards regimen, Cornaro simply "avoided extremes of heat and cold, overfatigue, late hours, sexual excesses and all violent passions of the mind;" he took moderate exercise in the open air, and his chief pleasures, were those obtained from literary and artistic study, from the contemplation of fine scenery, noble buildings, beautiful combinations of color and sweet music.

When Cornaro was within two years of fourscore, his diet was regulated in quality and quantity as follows: In four meals he took each day twelve ounces in all of solid food, consisting of bread (stale, of course, for he was not weak-minded), light meat, yolk of egg and soup; of liquid food other than pure water, he took fourteen ounces of light wine. Thus, his solid food, equally divided among four meals, amounted only to three ounces per meal, while he took per meal about three and one-half ounces, or as nearly as possible, one-third of a tumblerful of claret or some other wine of the kind.

At the age of eighty-three Cornaro wrote his treatise on "The advantage of a Temperate Life," adding later three other discourses on the same subject. His fourth and last discourse, which appeared in a letter addressed to Barbaro, patriarch of Aquileia, was written at the age of ninety-five. In this he says, "he finds himself still in possession of health and vigor, and in perfect command of all his faculties." According to some accounts, Cornaro lived to the age of one hundred and four. But comparing Cornaro's remarks in his discourses with the best information we have up to the time of his death, which appears to have occurred in 1566, it would seem that he was either in his ninety-ninth or one hundreth year when he died.

How much Cornaro's abstemious and ascetic ways must have had to do with his remarkable vitality, may be inferred from the fact that, having, when seventy years old, met with a terrible accident, by which his head and body were battered and a leg and an arm dislocated, he recovered—though the physicians had pronounced his injuries fatal—almost without medical treatment, and without any feverish symptoms.

The case of Thomas Wood, known as "the abstemious miller," is much the same. Wood had grown excessively corpulent and was suffering from a number of ailments, including violent rheumatism and frequent attacks of gout, when he read Cornaro's treatise. Gradually adopting the system there recommended, he soon found "his health established, his spirits lively, his sleep no longer disturbed by frightful dreams and his strength of muscle so far improved that he could carry a weight of a quarter of a ton at the age of fifty, whereas, at thirty, he had not been able even to move so much." He lost about one hundred and fifty pounds of his weight; but the exact amount is not known, as he was superstitiously unwilling to be weighed. Unfortunately, he was not content to follow Cornaro's experience, but tried absurd extremes of abstinence, absolutely going without liquid food altogether during the last sixteen years of his life.

His case, then, only shows what a burden is taken from the system when the quantity of food is reduced even far below what is commonly regarded as a moderate amount.

The records of no less than fifty-two centenarians were collected by a committee of the British Medical Association. Of the fifty-two, thirty-six are women. This may probably be attributed in large part to the comparative immunity that women enjoy from many risks to which men are exposed, but probably it is due not less to their greater temperance and to their freedom from the anxieties and heartburnings which attend men's struggles for influence, and even for maintenance. Medical men contend, however, that women also possess greater inherent vitality than men, the mortality of girls being less than that of boys, even during the first years of life, when the female is neither more temperate nor less ambitious than the male, and is exposed to as many dangers.

Of the sixteen men one only was single: ten of the thirty-six women were single; fifteen men and twenty-six women, then, among the centenarians, were married; but, naturally enough, of these forty-one a large number, all in fact, but five, were widowed. Three of the fifty-two were rich, nineteen poor, the rest in comfortable circumstances; nine were fat, twenty-three lean, eighteen medium; only eight were full-blooded; the rest average or pale. Forty had good digestion, which, after a hundred years, means a good deal. Most of the fifty-two have had good appetites, only two having appetites classed as actually bad; most of them have been through life moderate eaters ; twelve, however, have eaten large quantities of food. Only one is returned as a large eater of flesh food, and only one as a great consumer of alcoholic liquors preferrably beer. Only eight of all the number are classified as simply "irritable," but to these must be added five classed as "irritable and energetic."

As to smoking, thirty-two are non-smokers, seventeen smoke much (four of them being women), three moderately, and two a little ; only one chews ; thirty-seven avoid snuff. It is worthy of notice, that most of them were free from rheumatic and gouty troubles. The only man whose joints were stiff or deformed from such causes stated, in reply to questions as to his capacity and taste for strong drink, "I always drank as much as I could, and I always will."

When we take a number of cases such as these, in all clases of life, under many varied circumstances, it would appear, as though fivescore years were the natural or normal limit of human life ; and that when men die many years before that age is attained, the fault, apart from malignant disease or accident, has lain with themselves.

